# Nutritional status of adults with phenylketonuria on pegvaliase: A 15-month prospective study

**DOI:** 10.1016/j.ymgmr.2023.101015

**Published:** 2023-10-20

**Authors:** Krista Viau, Leslie Martell, Ann Wessel, Fran Rohr, Suzanne Hollander, Melissa S. Putman, Stephanie Sacharow

**Affiliations:** a Division of Genetics and Genomics, Boston Children's Hospital, 300 Longwood Ave., Boston, MA 02115, United States of America; b Department of Clinical Nutrition, Boston Children's Hospital, 300 Longwood Ave., Boston, MA 02115, United States of America; c Met Ed, Boulder, CO, 80302, United States of America; d Division of Endocrinology, Boston Children's Hospital, 300 Longwood Ave., Boston, MA 02115, United States of America; e Division of Endocrinology, Massachusetts General Hospital, 55 Fruit St., Boston, MA 02114, United States of America; f Department of Pediatrics, Harvard Medical School, Boston, MA 02115, United States of America

**Keywords:** Phenylketonuria, Pegvaliase, Nutritional status, Protein intake, Body composition, Eating behaviors

## Abstract

**Background:**

Pegvaliase has allowed many adults with phenylketonuria (PKU) to achieve acceptable blood Phe control while eating an unrestricted diet. However, little is known about potential differences in nutritional status and eating behaviors after transitioning from a phenylalanine (Phe)-restricted to an unrestricted diet. Here we assessed change in nutritional status in adults with early-treated PKU who were consuming a Phe-restricted diet (intact protein ≤0.8 g/kg/day) prior to starting pegvaliase.

**Methods:**

A 15-month, prospective, longitudinal study to assess change in anthropometrics, dietary intake, laboratory indices of nutritional status, bone mineral density (BMD), body composition, measured resting energy expenditure (REE), and eating behaviors between baseline and Month 15.

**Results:**

Eleven adults (*n* = 7 female) aged 19.5–52.9 years completed the study. Six participants had a substantial blood Phe reduction (responders) and five participants had a modest blood Phe reduction (partial responders) by Month 15. Intact protein intake increased by an average of 49.4 g/day and 26.7 g/day in responders and partial responders, respectively. Plasma concentrations of most vitamins, minerals, and essential fatty acids assessed were normal, though micronutrient intakes decreased as participants decreased or discontinued PKU medical food(s). Responders had a more variable change in body mass index (BMI) and lean mass index (LMI) compared to partial responders, though there were no clear trends in BMD or body composition changes. Total protein intake was positively correlated with LMI. Responders, but not partial responders, self-reported increased in enjoyment of food and decreased food neophobia, uncontrolled eating, and emotional eating.

**Discussion:**

Participants transitioning to an unrestricted diet while on pegvaliase maintained adequate nutritional status overall with no clinically significant changes in cardiovascular or glycemic markers. Responders reported improvements in eating behaviors, including reduced food neophobia, uncontrolled eating, and emotional eating, and increased enjoyment of food. There were no consistent trends in BMD, body composition, or BMI changes. A larger sample size and longer follow-up period are needed to further assess potential changes.

## 1 Introduction

Phenylketonuria (PKU) is an inherited disease of phenylalanine (Phe) metabolism caused by a deficiency of the hepatic enzyme phenylalanine hydroxylase. PKU is treated with lifelong dietary and/or pharmacological interventions to reduce blood Phe concentrations with a target of 120–360 μmol/L [1,2]. A tightly controlled Phe-restricted diet is effective at lowering blood Phe levels to prevent and/or reduce neurocognitive sequelae. However, for most, it becomes increasingly challenging to maintain the diet after childhood due to the degree of dietary restriction required for disease management and the necessity for Phe-free medical food(s) to meet total protein needs [3,4]. Related barriers to dietary adherence include access to and palatability of expensive medical foods and low-protein foods as well as the immense logistical and social challenges in following the diet. The need for non-dietary treatment(s) is well recognized, and a variety of pharmaceutical options have emerged in the research landscape, with a few now being clinically available.

Pegvaliase is an enzyme substitution therapy that has allowed many adults with PKU to achieve acceptable blood Phe control while eating an unrestricted diet [2]. However, pegvaliase therapy comes with several challenges, including a high likelihood of adverse event(s), risk of anaphylaxis, and an often lengthy and unpredictable time to response. In a cross-sectional study of individuals naïve to pegvaliase, authors reported that potential adverse events with pegvaliase were perceived as acceptable in exchange for reduced disease burden, improved blood Phe concentrations, and better quality of life [5].

Removal of the arduous Phe-restricted diet not only lessens disease burden but may also confer nutritional benefits. Studies have shown increased risk for nutritional deficiencies when following a Phe-restricted diet, particularly for individuals who take less than the prescribed amount of medical food [[6], [7], [8], [9]]. It may be easier for individuals to consume a well-balanced diet if they do not have to rely on medical foods and high carbohydrate, low protein foods as primary sources of energy. However, for many who have acclimated to a highly restrictive diet, there may be challenges in accepting new foods, such as fear of trying new food (food neophobia), guilt consuming previously forbidden foods, and poor perceived palatability and/or tolerance of high protein foods.

Here we examined the change in the nutritional status of adults over 15 months of treatment with pegvaliase. Specifically, this study aimed to describe changes in anthropometrics, dietary intake, laboratory indices of nutritional status, bone mineral density (BMD), body composition, and resting energy expenditure (REE) in adults consuming a Phe-restricted diet at baseline.

## 2 Methods

### 2.1 Study design

Participants were enrolled in a 15-month prospective, longitudinal study conducted between April 2019 and December 2022. Patients were identified and recruited through the Division of Genetics and Genomics at Boston Children's Hospital (BCH) and surrounding metabolic clinics. The BCH Institutional Review Board approved the study protocol, and written informed consent was obtained from all participants. Inclusion criteria included (1) adults aged 18–65 years, (2) diagnosis of PKU, (3) following a Phe-restricted diet with or without medical food (i.e., intact protein intake <0.8 g/kg/day) within 30 days of enrollment, and (4) within ±90 days of starting treatment with pegvaliase at enrollment. Individuals were excluded if currently pregnant or lactating, as nutritional needs differ.

All diet and pegvaliase prescriptions were determined by the participants' metabolic healthcare team. Study assessments included anthropometry (height and weight), fasting venous blood draw, dietary assessment, dual-energy x-ray absorptiometry (DXA) scan, indirect calorimetry (IC), review of medications/supplements, and questionnaires. All assessments were completed at Month 0 (baseline), Month 9 (±14 days), and Month 15 (±14 days) apart from DXA and indirect calorimetry, which were only performed at Months 0 and 15. The study was conducted in the Experimental Therapeutics Unit (ETU) or Metabolism Clinic at BCH. Due to the COVID-19 pandemic, all nutrition assessments were conducted remotely after March 2020, and the remaining assessments were conducted in-person as described above. However, three participants completed the Month 9 visit remotely with no in-person assessment due to the restrictions on elective research procedures at that time (May–June 2020).

### 2.2 Dietary assessment

#### 2.2.1 Three-day food record

Participants recorded food and beverage intake for three consecutive days prior to the study visit, including two weekdays and one weekend day. A study dietitian reviewed food records for accuracy and completeness. All food records were analyzed using MetabolicPro Diet Analysis software (www.metabolicpro.org).

#### 2.2.2 Food frequency questionnaire

Participants completed the “Food Frequency Questionnaire—a dietary assessment tool designed to help you manage your protein intake when following a protein-restricted diet” (© 2012 BioMarin Pharmaceutical Inc., Novato, California) to evaluate typical daily protein intake over the last month. The FFQ was previously evaluated in a pediatric and adult PKU population [10] and measures typical protein intake across 13 food categories: medical foods, special low protein foods, vegetables, fruits, grains, snack foods, beans, nuts, dairy, cheese, eggs, meat, and fast food/restaurant foods.

### 2.3 Eating behavior questionnaires

Participants were read a standard set of instructions and completed the following four questionnaires in the same order: Epicurean Eating Tendencies (EET) scale; modified, 9-item Food Neophobia Scale (FNS); Three Factor Eating Questionnaire Revised-18 (TFEQ-R18); and Emotional Eater Questionnaire (EEQ).

The EET scale measures one's aesthetic appreciation for food, including both sensory aspects and the symbolic value of food [11]. Raw scores range from 7 to 49 with higher scores indicating more prominent Epicurean eating tendencies.

The modified FNS measures aversion to trying new foods. The original 10-item scale was developed for and tested in healthy adults [12], and was later adapted to a 9-item scale for children with PKU [13]. Raw scores range from 9 to 63 with lower scores reflecting increased food neophobia [13].

The TFEQ-R18 assesses the concept of dietary restraint and includes three domains measuring cognitive restraint, uncontrolled eating, and emotional eating. The 18-item version of the TFEQ was revised from the original 51-item version [14]. While developed for populations with obesity, it has also been evaluated in general populations [15]. Raw scores range from 18 to 72 with higher scores indicating more of the behavior. Considering participants were consuming a Phe-restricted diet at baseline, one question in the uncontrolled eating domain was changed from “When I smell a sizzling steak or a juicy piece of meat, I find it very difficult to keep from eating…” to “When I smell a delicious food…”.

The EEQ is a 10-item questionnaire designed to evaluate the extent to which emotions affect eating behavior in adults with overweight and obesity. Raw scores range between 0 and 30 with higher scores indicating more emotional eating behaviors [16].

### 2.4 Biochemical assessment

All analyses were performed on venous blood samples collected after an overnight fast (≥8 h). Laboratory analyses included plasma amino acids, visceral proteins (prealbumin and albumin), plasma fatty acid profile, vitamin/minerals (vitamins A, E, and 25-hydroxy vitamin D (25(OH)D) using liquid chromatography-tandem mass spectrometry (LC-MS), red blood cell (RBC) folate, serum iron, ferritin, transferrin saturation, magnesium, methylmalonic acid (MMA), selenium, and zinc) and indices of cardiovascular and glycemic status (triglycerides (TG), total cholesterol (TC), high-density lipoprotein cholesterol (HDL—C), and low-density lipoprotein cholesterol (LDL-C), and hemoglobin A1c (HgA1c) using an NGSP-certified instrument). Laboratory analyses were conducted at Boston Children's Hospital apart from serum MMA and plasma fatty acid profile, which were analyzed at Children's Hospital Colorado and Mayo Clinic, respectively.

### 2.5 DXA

Areal BMD (g/cm^2^) of the total hip, femoral neck, and lumbar spine in the posterior-anterior (PA) projection was assessed using DXA (Horizon A, Hologic Inc., Bedford MA). Whole body scans were obtained to provide body composition measures, including fat mass (%), lean body mass (g), and android:gynoid fat mass ratio. Fat mass index (FMI) was calculated as fat mass (kg)/height (m)^2^ and lean mass index (LMI) was calculated as lean mass (kg)/height (m)^2^. FMI and LMI provide an assessment of body composition after controlling for differences in height. Age-, gender-, and race-specific BMD *Z*-scores were derived using the manufacturer's normative database.

### 2.6 Resting energy expenditure

Indirect calorimetry (IC) was offered to participants as an optional assessment. Resting energy expenditure was assessed using Vmax Encore by Carefusion. IC was performed in the morning after an overnight fast, and participants were instructed to limit physical activity prior to testing.

### 2.7 Pegvaliase response

While the term “response” is commonly used to describe significant blood Phe reduction due to treatment with pegvaliase, this differs from response to sapropterin dihydrochloride (pharmacological form of tetrahydrobiopterin). Individuals' response to sapropterin dihydrochloride is dependent upon on one's genotype and residual enzyme activity with approximately 50% of individuals with PKU responding [17,18]. In contrast, response to pegvaliase is genotype-independent, time-bound and related to one's immune response [19]. Thus, theoretically, anyone could respond to pegvaliase.

There are varying definitions of pegvaliase response, though efficacy is generally determined by a significant reduction in blood Phe concentrations with the ability to liberalize or normalize intact protein intake [20]. In this study, pegvaliase response was defined as having a blood Phe level of <360 μmol/L while consuming at least 40 g intact protein per day [21].

### 2.8 Statistical analysis

Statistical analyses were performed using Stata 17.0 (StataCorp, College Station, TX). Data were summarized using descriptive statistics: continuous variables were reported as mean ± standard deviation if normally distributed and median (interquartile range (IQR)) if not normally distributed, and categorical variables were reported as frequencies. Normality was assessed with the Shapiro-Wilk test.

An adjusted body weight [ideal weight + ((actual weight - ideal weight)*0.25)] was used to calculate energy and protein intakes per kilogram for participants with a body mass index (BMI) ≥30 kg/m^2^. The daily pegvaliase dose (mg/day) was calculated from the average weekly dose, as not all patients dose daily. The percentage difference in blood Phe, BMI, FMI, and LMI mass from baseline to Month 15 was calculated using the following equation: [((Month 15 value - baseline value)/baseline value)*100].

While this study was not powered to detect significant differences in outcomes from baseline to Month 15, the following exploratory analyses were conducted for the entire sample: 1) Spearman rank order correlations between total lean mass, REE, and protein intake (total and intact) and 2) paired *t*-test for normally distributed data comparing BMD and body composition from baseline to Month 15.

## 3 Results

A total of 12 adults consented to participate from three different metabolic centers and 11 completed the study. One male participant discontinued pegvaliase after the first 2.5 mg dose due to a hypersensitivity event and was removed from the study. Participants ranged from 19.5 to 52.9 years of age at enrollment, seven were female, and all were white (Table 1). At the Month 15 visit, participants had been on pegvaliase for an average of 75.2 ± 5.9 weeks (range 67.6–83.2 weeks). The average daily pegvaliase dose was 30 ± 12 mg/day (range: 11.4–40 mg/day) at Month 9 and 36.3 ± 20 mg/day (range: 5.7–60 mg/day) at Month 15.

**Table 1:** Participant demographics at baseline (presented as mean ± standard deviation or frequency).

	Female n = 7	Male n = 4	All *n* = 11
Age, years	34.5 ± 13.0	30.5 ± 15.3	35 ± 13.3
Body mass index, kg/m^2^^a^	31.6 ± 4.4	27.1 ± 2.9	30.0 ± 4.4
Healthy weight	0	1	1
Overweight	3	3	6
Obese	4	0	4
Education			
College	6	4	10
High School	1	0	1
Work Status			
Full time	5	2	7
Part time	2	1	3
Unemployed	0	1	1
Living Situation			
Living with significant other	1	1	2
Living with family	3	3	6
Living alone	3	0	3

a BMI categories (healthy weight = 18.5–24.9; overweight = 25.0–29.9; obesity ≥30 kg/m^2^).

Six out of 11 participants met our criteria for pegvaliase response during the 15-month observation period with four out of six participants responding by Month 9. More female participants responded by Month 15 (5 out of 7 females) compared to males (1 out of 4 males). On average, these six participants responded 33.7 ± 19.9 weeks (range: 9–58 weeks) after starting pegvaliase and had been on an efficacious pegvaliase dose for 43.4 ± 19.1 weeks (range: 21–68 weeks) prior to the Month 15 study visit. While the remaining participants did not meet our criteria for response, they also had a decrease in blood Phe with a concomitant increase in intact protein after self-liberalizing their diets over the study; thus, we labeled this group as “partial responders” as opposed to “non-responders”. At Month 15, partial responders were taking either 40 mg (*n* = 3) or 60 mg (*n* = 2) pegvaliase per day. One partial responder prescribed 40 mg pegvaliase/day reported poor compliance with pegvaliase prior to his Month 15 study visit.

**Fig. 1:**
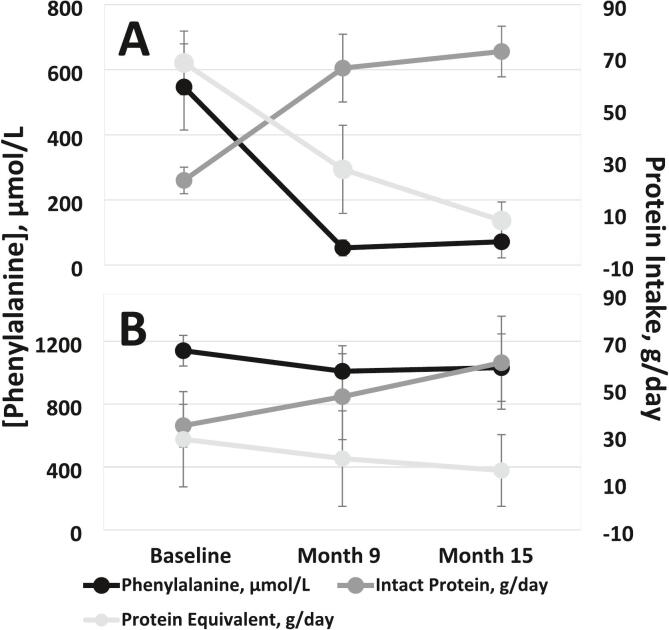
Change in blood phenylalanine and intake of intact protein and protein equivalents from Phe-free medical food(s) in responders (A) and partial responders (B). Data points reflect mean ± standard error.

At baseline, mean blood Phe was 547 ± 324 in responders and 1140 ± 218 in partial responders (Fig. 1). The median percentage change in blood Phe from baseline to Month 15 was −95% (IQR: −98 to −81%) in responders and − 18% (IQR: −39 to 16%) in partial responders. Five participants had one or more low blood Phe and six had one or more low blood tyrosine (Tyr) results. Apart from low blood Phe and Tyr, no deficiencies were identified in plasma essential amino acids, prealbumin, or albumin.

### 3.1 Protein intake

The two methods used to assess protein intake (food record and FFQ) showed similar results. Based on analysis of 3-day food records, participants consumed an average of 49.8 ± 41.0 g medical food protein/day and 27.8 ± 16.7 g intact protein/day at baseline, reflecting a moderate Phe-restricted diet. At baseline, all but one participant consumed some or all their prescribed medical food. Medical food intake decreased at each visit and intact protein intake increased by Month 15, regardless of pegvaliase response (Table 2). Five out of six responders exceeded the DRI for intact protein (0.8 g/kg/day) by Month 15. One responder was in the process of normalizing protein intake and consumed >50 g intact protein per day at Month 15 (0.55 g/kg/day).

**Table 2:** Energy and macronutrient intake calculated from three-day food records (presented as mean ± standard deviation).

	Month 0	Month 9	Month 15
**Responders**	n = 6	n = 6	n = 6
Energy, kcal/day	1830 ± 468	2153 ± 758	1897 ± 747
Per kg	24.4 ± 8.7	30.7 ± 12.4	26.3 ± 12.4
Total Protein, g/day	90.1 ± 23.8	92.3 ± 16.6	79.0 ± 23.1
Per kg	1.19 ± 0.35	1.29 ± 0.23	1.08 ± 0.36
Intact Protein, g/day	22.5 ± 12.6	65.6 ± 31.7	72.0 ± 23.7
Per kg	0.3 ± 0.2	0.9 ± 0.5	1.0 ± 0.4
Protein Equivalents^a^, g/day	67.6 ± 29.9	26.8 ± 41.4	7.05 ± 17.2
Per kg	0.9 ± 0.4	0.4 ± 0.6	0.1 ± 0.2
% kcal from total protein	20.6 ± 7.3	18.6 ± 7.4	17.1 ± 4.2
% kcal from carbohydrate	52.9 ± 2.4	48.4 ± 6.5	49.1 ± 4.8
% kcal from fat	26.5 ± 8.2	33.1 ± 8.3	33.9 ± 3.0
Fiber, g/day	19.2 ± 9.3	20.4 ± 4.6	17.8 ± 8.5
**Partial Responders**	*n* = 5	n = 4	n = 5
Energy, kcal/day	1963 ± 493	1943 ± 633	1909 ± 534
Per kg	25.8 ± 4.1	26.2 ± 6.8	26.7 ± 7.8
Total Protein, g/day	62.6 ± 40.2	66.7 ± 31.4	76.1 ± 34.5
Per kg	0.81 ± 0.47	0.89 ± 0.35	1.07 ± 0.50
Intact Protein, g/day	34.2 ± 20.2	46.5 ± 36.5	60.9 ± 44.0
Per kg	0.4 ± 0.3	0.6 ± 0.4	0.9 ± 0.7
Protein Equivalents^a^, g/day	28.4 ± 45.1	20.3 ± 40.5	15.2 ± 34.0
Per kg	0.4 ± 0.5	0.3 ± 0.6	0.2 ± 0.4
% kcal from total protein	11.7 ± 5.3	13.1 ± 2.7	15.0 ± 3.6
% kcal from carbohydrate	57.7 ± 4.3	55.4 ± 7.9	50.7 ± 6.7
% kcal from fat	30.6 ± 6.2	31.6 ± 8.9	34.3 ± 4.8
Fiber, g/day	16.1 ± 3.4	16.4 ± 4.1	13.8 ± 5.4

a Protein equivalents reflect Phe-free protein from PKU medical food(s).

Based on FFQ results at Month 15, responders consumed an average of 79.6 ± 39.3 g total protein, 6.0 ± 13.5 g medical food protein, and 73.6 ± 41.6 g intact protein with 42.3 ± 20% of intact protein coming from an animal protein (i.e., dairy, meat, or eggs; excludes whey protein supplements). In contrast, at Month 15 partial responders consumed an average of 55.7 ± 11.1 g total protein, 12.0 ± 26.8 g medical food protein, and 43.7 ± 18.5 g intact protein with 43.8 ± 25.8% of intact protein coming from an animal protein.

### 3.2 Micronutrient adequacy and cardiovascular health

At baseline, median dietary intakes of most vitamins and minerals assessed were adequate (≥100% of DRI): Vitamin A 154% (IQR 74–197%), Vitamin B12 163% (IQR 100–321%), calcium 123% (IQR 58–186%), folic acid 162% (IQR 92–203%), iron 160% (IQR 66–226%), magnesium 100% (IQR 66–188%), selenium 183% (IQR 110–253%), and zinc 125% (IQR 91–282%). Median intakes of Vitamin D 47% (IQR 27–147%) and Vitamin E 77% (IQR 41–129%) were less than the DRI at baseline. As the pegvaliase responders transitioned to an unrestricted diet and decreased medical food intake, median micronutrient intakes decreased with iron and vitamins E and D being below the DRI (data not shown).

Participants had normal median plasma concentrations of most vitamins, minerals, and essential fatty acids assessed (data not shown). However, 25-OH vitamin D concentrations were < 20 ng/mL in four participants (*n* = 1 at baseline, *n* = 3 at Month 9, and n = 1 at Month 15). Vitamin D intake from food was below the DRI for these participants. Additionally, six participants, of which five were responders, had low serum iron (*n* = 4 at baseline, *n* = 2 at Month 9, and n = 1 at Month 15), though ferritin and transferrin saturation were normal and iron intake from food was ≥100% DRI. In participants with low 25-OH vitamin D or serum iron at baseline or Month 9, values normalized by Month 15.

Most participants took one or more nutritional supplements during the study: eight participants reported a multivitamin and mineral supplement at 1–2 study visits, four reported a vitamin D supplement at 1–2 study visits, three reported a B12 supplement at 1–3 visits, two reported a fish oil/omega 3 supplement at 1–3 study visits, one reported a calcium supplement at two study visits, and one reported an iron supplement (in response to low serum iron) at one study visit.

There were no clinically significant changes in markers of cardiovascular and glycemic status over the study, and fasting markers of cardiovascular health were normal for most participants (data not shown). Abnormalities were noted in low HDL-C (*n* = 3 at all visits) and high TG (*n* = 2 at all visits). Family history of hyperlipidemia was not assessed.

### 3.3 Bone mineral density, body composition, and resting energy expenditure

**Fig. 2:**
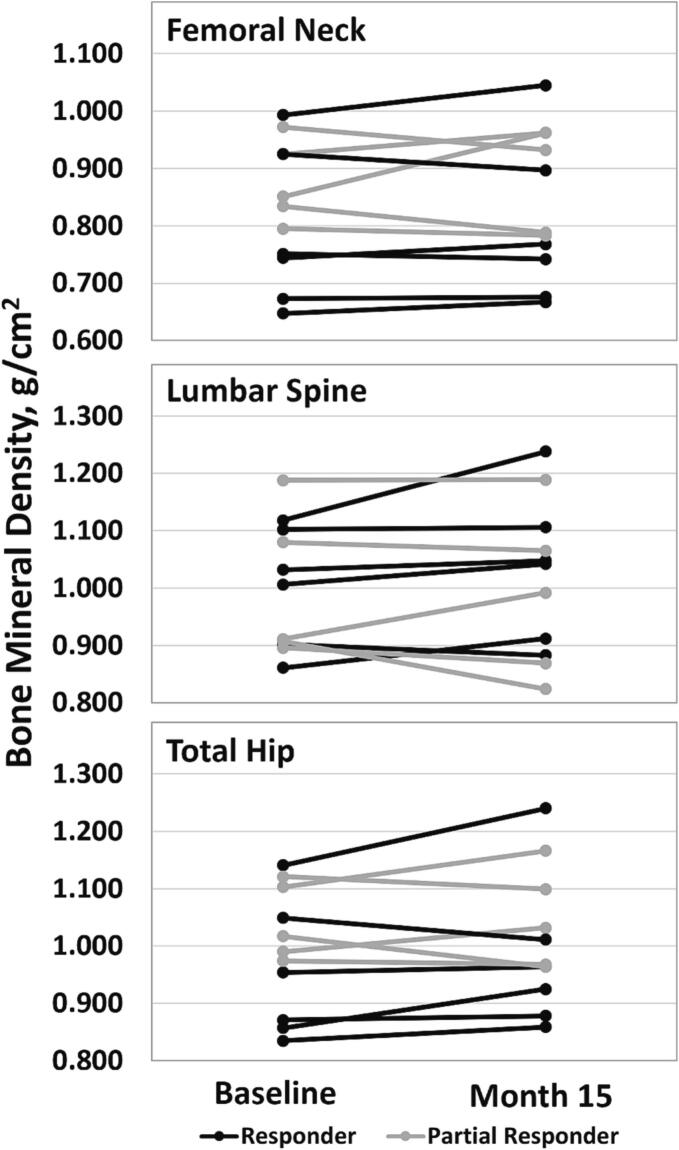
Bone mineral density (g/cm^2^) for femoral neck, lumbar spine (L1-L4), and total hip at Baseline and Month 15 in responders and partial responders. There were no significant differences between baseline and Month 15 (data not shown).

No participants had low BMD (*Z*-score ≤ −2.0) at the femoral neck, lumbar spine, or total hip at baseline. However, two partial responders had a decrease in lumbar Z-scores resulting in a low BMD Z-score at Month 15. Both participants had 25-OH vitamin D concentrations <30 ng/mL throughout the study and consumed <50% of their medical food prescriptions. One participant also routinely consumed inadequate energy and total protein intake (<0.5 g/kg/day). There were no significant differences in BMD at the femoral neck, lumbar spine, or total hip for the total sample (*n* = 11) from baseline to Month 15, *p* > 0.05 for all (Fig. 2).

**Fig. 3:**
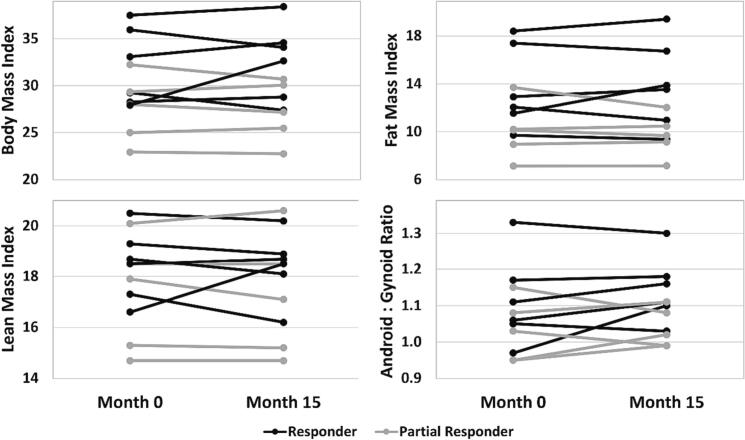
(Option 2) Body mass index (kg/m^2^), fat mass index (kg fat mass/m^2^), lean mass index (kg lean mass/m^2^), and android:gynoid ratio at Baseline and Month 15 in responders and partial responders. There were no significant differences between baseline and Month 15 (*p* > 0.05 for all).

Responders' percentage change in BMI from baseline to Month 15 was more variable than partial responders (range: −6.4 to +16.8% vs. -4.8 to +2.4%, respectively) with one responder experiencing a > 15% increase in BMI (Fig. 3). Responders' percent change in FMI and LMI ranged from −9.1% to +20.3% and − 6.4% to +11.4%, respectively. Partial responders had little change in LMI (<5% difference), though the difference in FMI ranged from −12.6% to +2.6%. All participants had an increased android:gynoid ratio at baseline and Month 15 indicating abdominal obesity [22]. There were no significant differences in BMI or body composition measures between baseline and Month 15, *p* > 0.05 for all (Fig. 3). In all participants, total protein intake (g/day) was positively correlated with LMI (rho = 0.59, *p* = 0.004). In contrast, there was no significant correlation between intact protein intake (g/day) and LMI (*p* = 0.08).

Indirect calorimetry was performed on eight participants (*n* = 4 responders and n = 4 partial responders) at Months 0 and 15. Pegvaliase responders had an average increase in resting energy expenditure of 97 ± 184 kcal/day (range − 78 to 261) and partial responders had an average decrease of 3 ± 51 kcal/day (range − 48 to 67). REE was strongly correlated with grams of total lean body mass (rho = 0.73, *p* = 0.0007).

### 3.4 Eating behavior questionnaires

Partial responders, on average, showed minimal change on questionnaires assessing food neophobia, Epicurean eating tendencies, uncontrolled eating, cognitive restraint, and emotional eating over 15 months of treatment with pegvaliase (Table 3). In contrast, as responders transitioned to an unrestricted diet between baseline and Month 15, the difference in questionnaire results suggest a decrease in food neophobia (FNS score increased by 8.6 ± 6.7; range: 2 to 18), increase in Epicurean eating tendencies (EET score increased by 8.8 ± 11.1; range: −4 to 26), decrease in uncontrolled eating (TFEQ-18 uncontrolled eating sub-score decreased by −5 ± 3.6; range: −9 to −2), and decrease in emotional eating (TFEQ-18 emotional eating sub-score decreased by −0.8 ± 1.6; range: −3 to 1; and EEQ score decreased by −5.2 ± 7.2; range: −16 to 4).

**Table 3:** Raw questionnaire scores (mean ± standard deviation) for responders and partial responders.

	Month 0	Month 9	Month 15	Range of possible scores
**Responders**	n = 5	n = 5	n = 5	
Food Neophobia Scale Score^a^	40.2 ± 11.1	52.0 ± 6.0	48.8 ± 8.0	9–63
Epicurean Eating Score^b^	27.0 ± 6.1	35.2 ± 5.5	35.8 ± 7.3	7–49
TFEQ-R18 Sub-score: Uncontrolled eating^b^	22.0 ± 5.0	18.2 ± 4.6	15.6 ± 5.2	9–36
TFEQ-R18 Sub-score: Cognitive restraint^b^	15.0 ± 2.3	15.2 ± 4.0	14.4 ± 3.8	6–24
TFEQ-R18 Sub-score: Emotional eating^b^	7.0 ± 3.5	6.8 ± 2.4	6.2 ± 3.7	3–12
Emotional Eating Questionnaire Score^b^	11.3 ± 7.4	8.6 ± 5.3	6.4 ± 4.5	0–30
**Partial Responders**	n = 5	n = 5	n = 5	
Food Neophobia Scale Score^a^	45.6 ± 8.3	39.8 ± 14.2	42.0 ± 12.3	9–63
Epicurean Eating Scale Score^b^	33.0 ± 5.8	30.4 ± 9.1	33.4 ± 8.2	7–49
TFEQ-R18 Sub-score: Uncontrolled eating^b^	18.0 ± 7.6	17 ± 9.1	17.6 ± 5.3	9–36
TFEQ-R18 Sub-score: Cognitive restraint^b^	10.2 ± 0.4	11 ± 1.6	13.0 ± 4.3	6–24
TFEQ-R18 Sub-score: Emotional eating^b^	7.0 ± 3.2	7.6 ± 2.4	7.0 ± 1.6	3–12
Emotional Eating Questionnaire Score^b^	15.4 ± 6.5	16.0 ± 6.2	13.0 ± 5.0	0–30

FNS, Food Neophobia Scale; TFEQ, Three Factor Eating Questionnaire Revised 18-item version; EEQ, Emotional Eating Questionnaire.

a FNS score: higher scores reflect less food neophobia.

b EET, TFEQ-R18, and EEQ scores: higher scores reflect more of the behavior.

## 4 Discussion

Approximately half (6/11) of participants responded to pegvaliase and transitioned to an unrestricted diet over the 15-month study. The remainder of participants demonstrated a modest Phe reduction while showing increased protein tolerance (Fig. 1). Pegvaliase is an enzyme substitution therapy comprised of pegylated phenylalanine ammonia lyase, a non-human enzyme, and is known to induce an immune response that contributes to delayed efficacy [19]. As there is a variable and unpredictable time to pegvaliase response, it is not unexpected to have some participants awaiting efficacy at Month 15.

Six of the participants (four responders) in this study were previously reported in Sacharow et al., 2020 [23]. That paper defined pegvaliase response as achieving a > 50% decrease in blood Phe with blood Phe ≤360 μmol/L. In the current study, pegvaliase response was defined as having a blood Phe level of <360 μmol/L while consuming at least 40 g intact protein per day [21]. Two responders in the current study had not yet responded to pegvaliase at the time of the Sacharow publication. While approximately 30% of patients were termed non-responders, nearly every patient (24/26) had at least a 30% decrease in blood Phe concentrations after starting pegvaliase [23]. In the current study, partial responders self-liberalized their diets to contain more intact protein and less medical food protein and 3/5 still had a reduction in blood Phe from baseline to Month 15 (Fig. 1), suggesting some therapeutic benefit of pegvaliase. One participant with an increase in blood Phe reported poor adherence with pegvaliase prior to the Month 15 visit.

At baseline, responders had lower blood Phe levels and were eating less intact protein and more medical food protein compared to partial responders. Timing of response is related to one's immune response, and there are generally no other known predictors of time to response at therapy initiation [19,24]. However, in this sample, responders started the study on a more restricted diet, which may suggest that they responded within the 15-month period, in part, because they were closer to the blood Phe blood threshold for efficacy at baseline. There may also be a group difference in treatment adherence to injection and diet recommendations during the 15 months on pegvaliase between those who began the study on a stricter low Phe diet and those who were less able to adhere to a Phe restriction.

Both groups had similar trends in nutrient intakes from baseline to Month 15, as all participants, whether advised or not, increased intact protein and decreased intake of medical foods over the study. Energy intakes (kcal/day) were relatively stable in both groups. At Month 15, mean intake of animal proteins comprised approximately 40% of total protein intake for both groups, indicating that the partial responders were also routinely consuming high protein foods. As intact protein intake increased, participants consumed more energy from fat and less from carbohydrates. This was also shown by McWhorter et al., (2022) when comparing diet composition of individuals with PKU treated with a traditional diet versus pegvaliase [25]. Participants' mean fiber intake decreased slightly, which may reflect decreased intake of grains, fruits, and/or vegetables. Overall, micronutrient intakes decreased as participants discontinued or decreased medical food. At Month 15, suboptimal dietary intake of micronutrient(s) was more prevalent in the responders who were consuming more medical food at baseline. This suggests the need for continued dietary assessment from a dietitian and anticipated benefit of vitamin/mineral supplementation if intake is inadequate as patients adapt to an unrestricted diet [26].

Most participants had adequate protein, micronutrient, and fatty acid nutriture based on serum assessments as well as normal lipid profile and HbA1c. Similar to a previous publication [27], we did not identify any deficiencies in essential plasma amino acids or visceral proteins when participants had low blood Phe (<30 μmol/L). The most common micronutrient deficiency was 25-OH vitamin D. Participants were living in a high latitude region, which may have contributed to the low vitamin D results. We did not identify any patterns of low 25-OH vitamin D with the month of the year. All low 25-OH vitamin D results (<20 ng/mL) occurred in individuals consuming inadequate vitamin D and no medical food, which is typically supplemented with vitamin D. Guidelines for the management of PKU with pegvaliase recommend monitoring biomarkers of nutritional status at the start of pegvaliase and every 6–12 months as indicated [26].

There were no consistent trends in BMD changes from baseline to Month 15. At baseline, no participants had low BMD *Z*-scores. The two partial responders with a low lumbar BMD Z-score at Month 15 had nutritional risk factors, including low 25-OH vitamin D, no medical food, and inadequate calcium and vitamin D intake. One of the two participants was also noted to consume inadequate energy and total protein intake.

Responders had a wider range of BMI, FMI, and LMI changes over 15 months despite both groups increasing intact protein. Prevalence of overweight and obesity has been reported to be higher in adults with PKU compared to controls [28,29]. All responders (*n* = 6) had overweight or obesity at baseline and two had a decrease in BMI and three had a decrease in FMI from baseline to Month 15. Removal of dietary Phe restriction provides an opportunity to consume a well-balanced diet and support achievement and/or maintenance of a healthy weight. However, as patients adapt to an unrestricted diet, barriers such as acceptance of high protein foods, unfamiliarity with cooking methods, and cost of foods that may have been previously covered by insurance may negatively impact food choices and/or contribute to over-reliance on highly processed foods and/or restaurant foods with large portions. Additionally, some patients may experience a period of food exploration after pegvaliase response during which diet quality may suffer. In a cross-sectional study of adults after a mean of 4.9 years on pegvaliase, diet quality was comparable to US adults based on NHANES 2015–2016 data [27], though the diet quality for both groups was not optimal. Education on healthy food choices as patients adapt to an unrestricted diet remains an important component of care [26].

As has been previously demonstrated, resting energy expenditure was strongly correlated with total lean mass [30]. Thus, strategies to increase and/or maintain lean mass may be beneficial to support a healthy weight in adults with PKU. Two studies reported a positive association between intact protein intake and lean body mass in children and adults with PKU following a Phe-restricted diet [31,32]. In this study, total protein intake was significantly correlated with grams lean body mass. While there were no significant changes in BMD or body composition from baseline to Month 15, this may be due to the small sample size. Additional studies are needed with a larger sample to further assess BMD, BMI, and body composition after a longer and, ideally, consistent period since transitioning to an unrestricted diet.

Responders self-reported improved eating behaviors from baseline to Month 15 with less food neophobia, emotional eating, and uncontrolled eating and more enjoyment and appreciation of food. There are a variety of potential reasons for these results, including improved mental health from reduced blood Phe concentrations, greater satiety while consuming additional intact protein, and/or improvement in overall quality of life from removing dietary protein restrictions, such as improvement in social aspects of eating. In contrast, there was little change in responses from partial responders even though most self-liberalized their diets. This may suggest a sense of guilt from self-liberalizing against recommendations and/or lack of the abovementioned benefits of pegvaliase response. Reduction of negative eating behaviors, such as uncontrolled eating, may have lasting benefits for overall health and weight maintenance in adults with PKU.

## 5 Limitations

There are several limitations to this study. The study had a small sample size with limited racial and ethnic diversity and over-representation of overweight and/or obesity; therefore, the results may not be generalizable to a broader PKU population. It is also well known that self-reported food intake data are subject to under- and over-reporting.

Other limitations include variable duration of therapy with pegvaliase and time on an unrestricted diet. Participants' titration schedules varied depending on individual tolerance and clinician experience and approach, and/or logistical issues such as clinic schedule and patient availability. The length of the 15-month study was inadequate to allow some participants time to respond to therapy. Some of the partial responders reported here had a more complete response to pegvaliase after the study completion. Additionally, there was self-described dosing non-adherence in one participant, and it may not have been captured for others due to lack of a dosing diary. An additional limitation was changing dose options over time. The label expansion for pegvaliase for a 60 mg dose trial for 16 weeks was approved in October 2020. Since the study began in April 2019, some individuals may not have dose escalated to 60 mg with enough time to allow for demonstration of response. In the Phase 3 Prism studies, 19% of participants required this dose level to achieve efficacy [33].

## 6 Conclusions

This study demonstrated that adults transitioning to an unrestricted diet while on pegvaliase maintained adequate protein, micronutrient, and fatty acid nutriture. Responders reported improvements in eating behaviors including reduced food neophobia, uncontrolled and emotional eating, and increased enjoyment of food. The ability to consume unrestricted diet with acceptable blood Phe control has the potential to optimize diet quality and improve overall health. There were no consistent trends in BMD, body composition, or BMI changes. A longer follow-up period and larger sample are needed to further assess potential changes. Additionally, most participants in this sample consumed a moderate Phe restriction at baseline. Individuals consuming a stricter low Phe diet may experience more significant changes in nutritional status and/or body composition.

## Funding

This study was supported by an investigator-initiated research grant from 10.13039/100008484BioMarin Pharmaceutical, Inc.

## Declaration of Competing Interest

None.

## Data Availability

The data that has been used is confidential.
